# Protective autophagy decreases lorlatinib cytotoxicity through Foxo3a-dependent inhibition of apoptosis in NSCLC

**DOI:** 10.1038/s41420-022-01027-z

**Published:** 2022-04-22

**Authors:** Conghua Lu, Rui Yu, Chong Zhang, Caiyu Lin, Yuanyao Dou, Di Wu, Yonghong Pan, Tao Peng, Huan Tang, Rui Han, Yong He

**Affiliations:** 1 Department of Respiratory Disease, Daping Hospital, Army Medical University, 400042 Chongqing, China; 2 Department of Ultrasound, The First Affiliated Hospital of Chongqing Medical University, 400042 Chongqing, China

**Keywords:** Targeted therapies, Preclinical research

## Abstract

Lorlatinib is a promising third-generation anaplastic lymphoma kinase (ALK) tyrosine kinase inhibitor (TKI) that has been approved for treating ALK-positive non-small-cell lung cancer (NSCLC) patients with previous ALK-TKI treatment failures. However, the inevitable emergence of acquired resistance limits its long-term efficacy. A more comprehensive understanding of the acquired resistance mechanisms to lorlatinib will enable the development of more efficacious therapeutic strategies. The efficacy of chloroquine (CQ) in combination with lorlatinib in ALK-positive NSCLC cells in vitro and in vivo was assessed using CCK-8, colony formation, immunofluorescence staining, flow cytometry analysis, western blot analysis, and xenograft implantation. Here, we show that lorlatinib induced apoptosis and protective autophagy in ALK-positive NSCLC cells. However, the protective autophagy can gradually lead to decreased cytotoxicity of loratinib in ALK-positive NSCLC cells. Meanwhile, we found that the combination of lorlatinib and CQ, an inhibitor of autophagy, inhibited autophagy and promoted apoptosis both in vitro and in vivo, which sensitized cells to lorlatinib through the dephosphorylation of Foxo3a and promoted nuclear translocation, then activation of Foxo3a/Bim axis. Taken together, our results suggest that inhibition of protective autophagy might be a therapeutic target for delaying the occurrence of acquired resistance to lorlatinib in ALK-positive NSCLC patients.

## Introduction

Non-small cell lung cancer (NSCLC) patients who harbor chromosomal rearrangements of the anaplastic lymphoma kinase (*ALK*) gene are highly sensitive to small-molecule ALK tyrosine kinase inhibitors (TKIs) [1–3]. Standard treatment of patients with advanced ALK-positive NSCLC has recently shifted to sequential treatment with crizotinib, followed by increasingly potent second-generation ALK TKIs, including alectinib, brigatinib, and ceritinib [4–6]. However, although most patients derive clinical benefits from second-generation ALK-TKIs, acquired resistance invariably develops in some patients and leads to clinical relapse [7]. Lorlatinib is a third-generation ALK-TKI and an ATP-competitive small-molecule inhibitor that targets ALK, and it can overcome resistance to first- and second-generation ALK-TKIs, even including those mediated by the G1202R mutation of *ALK* [8, 9]. Unfortunately, despite the improved efficacy of lorlatinib, drug resistance still develops and the disease recurs, which have greatly limited the long-term efficacy of lorlatinib, especially in NSCLC [10]. Therefore, understanding the mechanisms leading to lorlatinib resistance is the key to the development of better therapeutic choices for treating patients using ALK-TKIs.

Recent studies have shown that the mechanisms underlying the acquired resistance to lorlatinib can be divided into ALK-dependent mechanisms, such as ALK mutations (G1202R/T1151M or L1196M/D1203N), and ALK-independent mechanisms, such as increased rates of epithelial-mesenchymal transition (EMT). Other resistance mechanisms driving acquired lorlatinib resistance are not fully clear [10, 11]. Meanwhile, our previous studies have shown that autophagy can be a potential pathway to TKI resistance, as inhibiting autophagy increases the sensitivity of TKI-resistant lung cancer cells to TKIs via induced apoptosis [12, 13]. In particular, previous studies have shown that crizotinib induces autophagy in multiple lung cancer cell lines, and inhibiting autophagy can suppress cell survival and promote crizotinib-induced apoptosis [13]. Nevertheless, the regulatory mechanism between protective autophagy and apoptosis remains not very clear.

To better exploit this phenomenon, we need to fully understand the routes of drug resistance and devise strategies to prevent or overcome ALK-TKIs resistance. Previous research has shown that autophagy was induced by TKI treatment and that autophagy played an important role in TKI resistance [12, 14]. However, whether lorlatinib can induce autophagy and the potential effects and mechanisms of lorlatinib-induced autophagy are unclear. In this study, we report that gradually increasing lorlatinib treatment induced autophagy and reduced sensitivity to lorlatinib in NSCLC cell lines. Moreover, we identified a set of elements that induce autophagy during lorlatinib treatment. Using in vitro and in vivo assays, we demonstrated the effects of lorlatinib-induced apoptosis and protective autophagy in NSCLC cells, whereas the protective autophagy can lead to decreased cytotoxicity of loratinib. Further work showed CQ could restrain protective autophagy and promote apoptosis of lorlatinib through activation of the Foxo3a/Bim axis. Our study first reveals a promising therapeutic strategy for sensitizing loratinib and delaying lorlatinib resistance in the future.

## Results

### Lorlatinib effectively inhibited the proliferation of ALK-positive NSCLC cells by promoting apoptosis

**Fig. 1:**
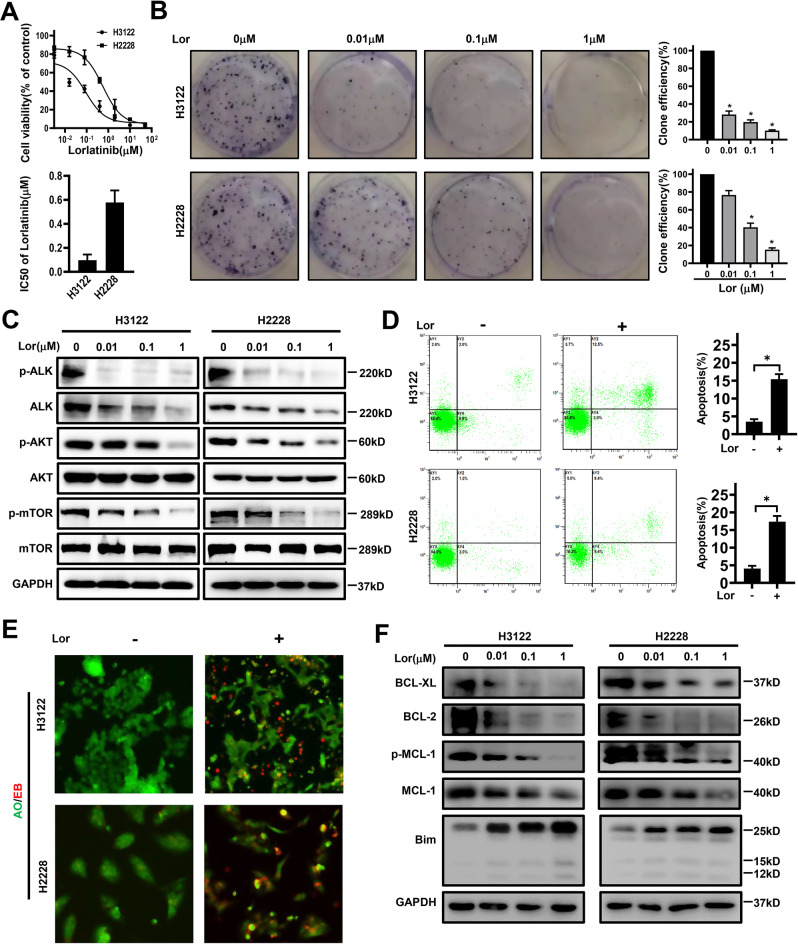
Lorlatinib reduces the viability of lung cancer cells. **A** CCK-8 assay using H3122 and H2228 cells treated with the indicated concentrations of lorlatinib for 48 h. **B** Colony-formation assay in cells treated with various concentrations of lorlatinib. **C** Western blot showed the expression of phosphorylation/total ALK, AKT, and mTOR in lorlatinib-treated H3122 and H2228 cells. **D** Cells were treated with lorlatinib (10 nM). The ratio of apoptotic cells was measured by Annexin V-FITC and propidium iodide (PI) staining. **E** AO/EB staining analyzed lorlatinib-induced apoptosis. **F** Cells were treated with the indicated concentrations of lorlatinib for 48 h. And after that, western blot analysis was performed on the Bim, BCL-2, MCL-1, P-MCL-1, and BCL-XL expression levels. Cells indicated that experiments were performed in triplicates and the results are shown as mean ± S.E.M. Lor lorlatinib. *P < 0.01 compared with control.

In order to verify the inhibitory ability of lorlatinib in ALK-positive NSCLC cells, we first observed the proliferation of H3122 and H2228 cells under lorlatinib treatment. The structure of lorlatinib is shown in Fig. S1A. Next, H3122 and H2228 cells were treated with various concentrations of lorlatinib for 48 h. Cell viability was assessed by CCK-8 assay. Growth in the two NSCLC cell lines was clearly inhibited by lorlatinib (Fig. 1A). Meanwhile, the long-term proliferation ability of H3122 and H2228 cells was evaluated by colony-formation assay. Under the pressure of lorlatinib for 14 days, colony numbers were reduced in a concentration-dependent manner (Fig. 1B). As is well known, the AKT/mTOR pathway is one of the important signaling pathway downstream of ALK, and it plays an important role in cell proliferation and survival in cancer [15]. Therefore, we analyzed the key protein activity in the AKT/mTOR pathway by western blot. The results showed that lorlatinib induced a rapid, dose-dependent decrease in total ALK and phosphorylation of AKT and mTOR (Fig. 1C). Moreover, morphological examination clearly demonstrated morphological changes in these cells, and the number of viable cells also decreased in a concentration-dependent manner (Fig. S1B). These results demonstrated that lorlatinib effectively inhibited the proliferation of ALK-positive NSCLC cells.

To further distinguish the morphological changes in these cells from apoptotic or necrotic changes, several other assays were performed. Based on the results of CCK-8 assays, the working concentration of lorlatinib was set at 10 nM, as this concentration did not cause significant cell death. Flow cytometry was used to analyze the number of apoptotic cells after Annexin V-FITC and PI staining. Compared with the control group, treatment with lorlatinib significantly induced apoptosis in H3122 and H2228 cells (Fig. 1D). AO/EB staining was also used to further verify the effects of lorlatinib-induced apoptosis. Early apoptotic cells were stained green with yellow dots and blebbing was present in their cytoplasm, while late apoptotic cells were stained orange red with fragmented nuclei. Non-apoptotic cells were stained green. The phenomenon that more EB passes through damaged cell membranes, embeds in the nucleus DNA, and brighter orange red fluorescence accumulates in the nucleus was observed in the group treated with lorlatinib (Fig. 1E). Consistently, immunoblotting results showed that lorlatinib significantly increased the expression of the apoptosis-related protein Bim and decreased MCL-1, p-MCL-1, BCL-2, and BCL-XL in H2228 and H3122 cells (Fig. 1F). Taken together, our results demonstrated that lorlatinib significantly induced apoptosis in a dose-dependent manner in ALK-positive NSCLC cells.

### Lorlatinib modulates the autophagic flux levels of ALK-positive NSCLC cells

**Fig. 2:**
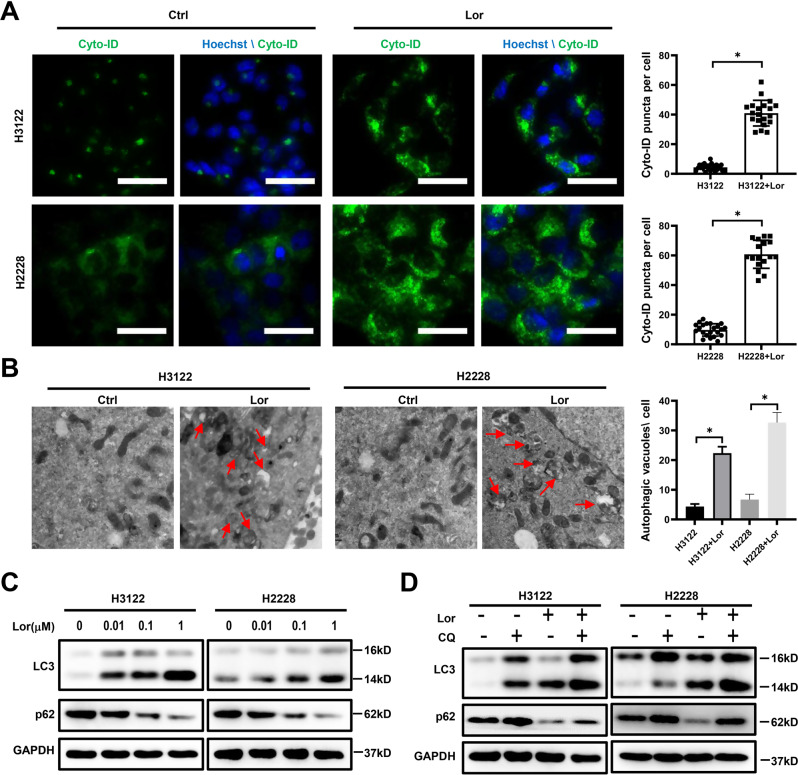
Lorlatinib induced autophagy in ALK-positive lung cancer cells. **A** Fluorescence photographs were shown the autophagosomes of H3122 and H2228 cells which were treated with lorlatinib (10 nM) for 48 h. Scale bar is 40 μm. **B** Transition electron microscopic images of cells treated with or without lorlatinib for 48 h. More autophagic vacuoles (AVs) were observed in the lorlatinib-treated group than in the control group. **C** H3122 and H2228 cells were treated with the indicated concentrations of lorlatinib for 48 h. And after treatment, western blot analysis was performed to measure the protein expression levels. GAPDH was included as a loading control. **D** H3122 and H2228 incubated with lorlatinib (10 nM) for 48 h, with or without treatment with CQ (10 μM) were analyzed for autophagy flux by western blot analysis. Ctrl control, Lor lorlatinib, CQ chloroquine. *P < 0.01.

To explore the potential mechanisms of induced drug sensitivity decreased, we next investigated whether lorlatinib treatment induced autophagy in ALK-positive NSCLC cells. We first observed the pattern when cells were exposed to a cyto-ID green detection reagent that selectively labeled accumulated autophagic vacuoles. More pre-autophagosomes, autophagosomes, and autolysosomes were visible after treatment with lorlatinib in H3122 and H2228 cells as compared to untreated cells (Fig. 2A). We further used transmission electron microscopy (TEM) to identify the accumulation of autophagosomes. Our TEM results also showed that autophagic vesicles were significantly increased in the lorlatinib group (Fig. 2B). Next, we assessed the expression of p62 and LC3 by western blot, as these are mainly protein markers of autophagy. Our western blot result showed that lorlatinib reduced the expression of p62 and increased LC3 in a concentration-dependent manner in H3122 and H2228 cells (Fig. 2C). These results indicated that treatment with lorlatinib could induce autophagy in ALK-positive NSCLC cells. To further confirm that, we assessed autophagy markers in H3122 and H2228 cells after blocking the autophagy flux with CQ (chloroquine diphosphate salt, which inhibits the integration of autophagosomes with lysosomes). The results showed that compared to lorlatinib treatment alone, the p62 levels were significantly increased under lorlatinib plus CQ combined treatment, and LC3 further increased. This indicated that lorlatinib induced high autophagic flux (Fig. 2D). Taken together, these results indicated that lorlatinib modulated the autophagic flux levels in ALK-positive H3122 and H2228 cells.

### Protective autophagy mediates lorlatinib desensitization in vitro

**Fig. 3:**
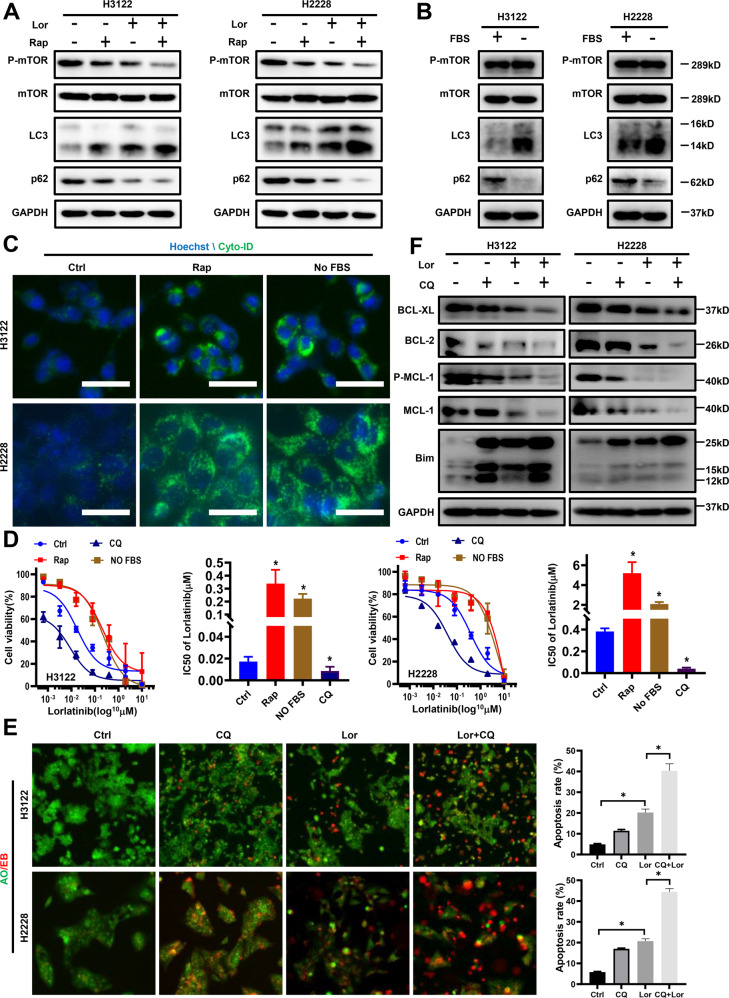
Lorlatinib-induced autophagy protects cells from apoptosis. **A** Cells were treated with lorlatinib (10 nM) with or without rapamycin (500 nM) for 48 h and observed changes in the content of LC3, p62, p-mTOR and total mTOR. **B** Western blot analysis of cells treated with or without fetal bovine serum (FBS) for 48 h. **C** Fluorescence photographs were shown the autophagosomes of H3122 and H2228 cells, which were treated with Rap (500 nM) or serum deprivation. Scale bar is 40 μm. **D** Cell viability CCK-8 assay for cells treated with the indicated concentrations of lorlatinib with or without CQ/ rapamycin/FBS for 48 h. *P < 0.01 compared with control. **E** Cells were treated with lorlatinib with or without CQ for 48 h, then stained with AO/EB. *P < 0.01. **F** Cells were treated by lorlatinib with or without CQ for 48 h. Western blot analysis was performed on the Bim, BCL-2, MCL-1, P-MCL-1, BCL-XL expression levels. GAPDH was included as a loading control. Lor lorlatinib, CQ chloroquine, Rap rapamycin.

We next used the pharmacological promotion of autophagy to explore whether autophagy induced desensitization to lorlatinib. Rapamycin or serum starvation has been reported as a common autophagy activator or method in in vitro assays [16]. Thus, we used rapamycin-treated H3122 and H2228 cells, and the LC3 and p62 expression levels were measured by western blot. The results showed that rapamycin induced autophagy, manifested by increased conversion of LC3I to LC3II and decreased expression of p62 protein, and these results were similar to those observed when H3122 and H2228 cells were serum starved (Fig. 3A–C and Fig. S2). Indeed, pharmacological induction of autophagy in cells with rapamycin or serum starvation resulted in decreased lorlatinib sensitivity. Meanwhile, we also found that treatment with CQ blocked the lorlatinib-induced autophagy flux and significantly increased lorlatinib sensitivity in both H3122 and H2228 cells (Fig. 3D). These results suggested that autophagy plays an important role in decreasing lorlatinib cytotoxicity.

We next further assessed the role of autophagy in lorlatinib-induced apoptosis. AO/EB staining was used after we used the lysosome autophagy inhibitor CQ plus lorlatinib, and these cells further induced apoptosis quite effectively (Fig. 3E). Moreover, further western blot analysis showed that the combination of CQ and lorlatinib produced sustained Bim elevation and more significant downregulation of MCL-1, P-MCL-1, BCL-2, and BCL-XL protein expression, as compared to a single treatment with either CQ or lorlatinib (Fig. 3F). Similar results were observed in H3122 and H2228 cells, which were treated with 3-MA (Fig. S3A–C). These results suggested that lorlatinib induced protective autophagy to protect cells from lorlatinib-dependent apoptosis.

### Lorlatinib promotes dephosphorylation of Foxo3a and its nuclear translocation

**Fig. 4:**
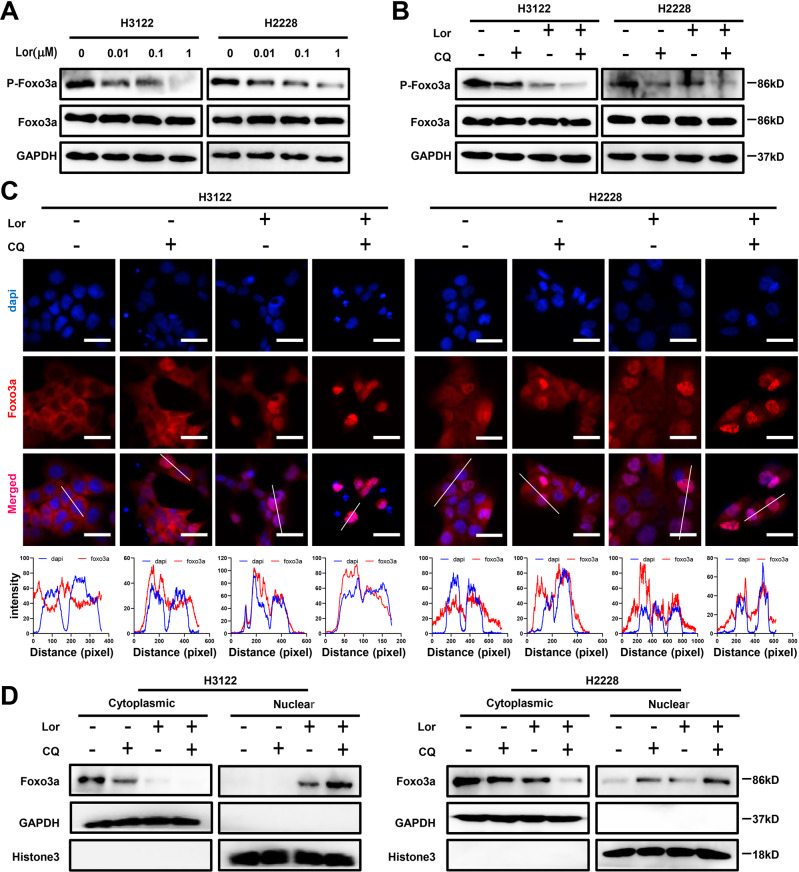
Lorlatinib affects FoxO3a phosphorylation and nuclear translocation. **A** Western blot analysis showed that the phosphorylation level of FoxO3a decreased with the increase of lorlatinib concentration. **B** Co-treatment with lorlatinib (10 nM) and CQ (10 μM) further decreased the phosphorylation level of FoxO3a. **C** Immunofluorescence staining showed that FoxO3a localized in the nucleus under lorlatinib treatment, while combined with CQ resulted in increased nucleus localization of FoxO3a. The nucleus was stained with 4′, 6-diamidino-2-phenylindole. Plots of pixel intensity along the white line from the top and bottom rows of images were drawn below each plot. **D** The nuclear and cytoplasmic protein fractions from H3122 and H2228 cells treated with lorlatinib and/or CQ were immunoblotted with the indicated antibodies. Western blotting showed that whether in H3122 or H2228 cells, using CQ to inhibit the autophagy produced by lorlatinib can induce FoxO3a to shift from cytoplasm to nucleus. GAPDH and Histone3 were included as a loading control. Lor lorlatinib, CQ chloroquine.

Previous studies have shown that phosphorylation of Foxo3a affects its shuttling between the nucleus and the cytoplasm, which then affects autophagy and cellular apoptosis [17, 18]. Therefore, we explored whether lorlatinib affected Foxo3a phosphorylation and nuclear translocation. western blot analysis showed that the phosphorylation level of Foxo3a decreased with the increasing concentration of lorlatinib (Fig. 4A). Co-treatment with lorlatinib and CQ further decreased the phosphorylation levels of Foxo3a, while the total amount of Foxo3a did not change (Fig. 4B). Next, we investigated whether this reduction in Foxo3a phosphorylation upon treatment with lorlatinib and CQ would further affect the nuclear translocation of Foxo3a. Immunofluorescence results showed that lorlatinib induced Foxo3a nuclear translocation, while lorlatinib and CQ acted together to further enhance its nuclear translocation (Fig. 4C). In order to further confirm the nuclear translocation of Foxo3a under the action of lorlatinib or/and CQ, we separated cytoplasmic and nuclear protein fractions and determined the expression of Foxo3a using western blot. The results showed that lorlatinib treatment increased the Foxo3a levels in the nucleus and decreased them in the cytoplasm, and when lorlatinib was combined with CQ, the effect was more significant (Fig. 4D). Taken together, these results indicated that the combination of lorlatinib and CQ could promote apoptosis through dephosphorylation of Foxo3a and by promoting its nuclear translocation.

### Combination of CQ with lorlatinib effectively inhibited the growth of H3122 xenografts

**Fig. 5:**
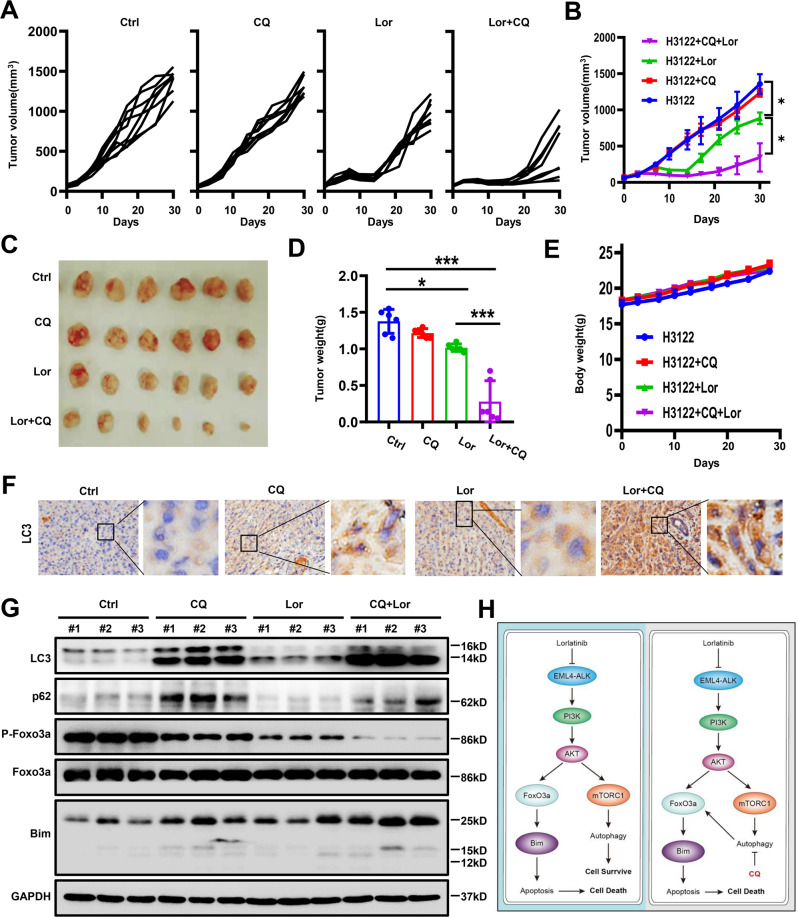
Inhibition of tumors in vivo by lorlatinib and CQ. **A**, **B** Tumor sizes are presented as the mean ± S.E.M. (*n* = 6); *P < 0.05. **C** Macroscopic appearance of H3122 xenografts treated with control, CQ, lorlatinib, and combined CQ/lorlatinib for 4 weeks. **D** Average tumor weight for each group was calculated. *P < 0.05. ***P < 0.001. **E** Body weight is presented as the mean ± S.E.M. (*n* = 6). **F** Representative images of IHC staining of LC3. **G** Whole-protein cell lysates were prepared randomly from three tumors per group for western blot to detect the indicated proteins. **H** The mechanism served to enhance the cytotoxicity of lorlatinib by inhibiting protective autophagy and then promoting Foxo3a nuclear translocation. Ctrl control, Lor lorlatinib, CQ chloroquine.

Based on the above findings, we next used a H3122 xenograft mouse model and further assessed the efficacy of the combination of the autophagy inhibitor CQ and lorlatinib in inhibiting xenograft tumor growth. As expected, administration of lorlatinib to H3122 xenograft mice resulted in slight tumor shrinkage, while treatment with CQ had little effect on tumor size. However, administration of a combination of CQ and lorlatinib resulted in significant tumor shrinkage, compared with lorlatinib alone (*P* < 0.05; Fig. 5A, B). Then, we compared the appearance and weight of tumor specimens. Consistent with tumor volume results, lorlatinib caused tumor shrinkage, and lorlatinib combined with CQ treatment showed more effective inhibition (Fig. 5C, D). During the entire treatment period, no significant weight loss was observed in mice treated with CQ and/or lorlatinib (Fig. 5E).

Through the observation that combined therapy was more effective than monotherapy in H3122 xenografts, we further explored the potential mechanism of this efficacy. The expression of LC3 in xenografts was determined using immunohistochemistry. Compared with any other group, the expression of LC3 was significantly increased in the co-treatment with lorlatinib and CQ group (Fig. 5F). Western blotting was used to detect protein expression in H3122 xenografts, and the results showed that phosphorylation of Foxo3a was strongly inhibited, Bim expression was significantly elevated, and LC3 was further significantly increased in mice treated with the combination compared with mice treated with CQ or lorlatinib alone (Fig. 5G). Collectively, these findings further suggested that the combination of CQ and lorlatinib enhanced the therapeutic efficacy in vivo, by inhibiting lorlatinib-induced autophagy and promoting apoptosis.

## Discussion

Drug resistance is the current limitation of ALK-TKI therapies, including lorlatinib [19]. To better avoid this possibility, we need to fully understand the routes of drug resistance and devise strategies to prevent or overcome them. In this study, we present the first evidence that lorlatinib-induced autophagy in ALK-positive NSCLC cells results in acquired resistance by itself. We used two cell lines to confirm that lorlatinib could induce autophagy and that this autophagy effect was protective in ALK-positive NSCLC cells. Previously, activation of survival promoting autophagy was found for therapeutic drugs used to treat cancer, and inhibition of autophagy can promote cell death [20]. In particular, previous reports have suggested that autophagy plays a role in EGFR-TKI osimertinib therapy for lung cancer [21–23]. For instance, autophagy can act to promote cancer progression by helping cancer cells survive stress and to induce carcinogenesis by influencing cell signaling or inhibiting intracellular apoptosis [24].

Reviewing the previous studies, the mechanism of autophagy regulating apoptosis is unclear. In this study, we began to explore the ways in which autophagy and apoptosis are related. We focused on the key molecule Foxo3a as it has been studied extensively in terms of apoptosis in recent years [25, 26]. We demonstrated that the inhibition of AKT leads to Foxo3a dephosphorylation and translocation into the nucleus, leading to apoptosis by inducing the expression of Bim. We have also been studies showing that inhibiting autophagy causes cells to increase Foxo3a activity, similar results have been confirmed in other studies [27]. Moreover, the mechanism of Foxo3a regulation also creates a link between autophagy and apoptosis that causes autophagy-inhibited cells to simultaneously be sensitized to apoptosis by upregulating BBC3/PUMA [28]. Consequently, we further explored the relationship between lorlatinib-induced autophagy and Foxo3a-regulated apoptosis.

Previous studies have found that the process of autophagy has been proposed as a good target for reversion of the resistance to ALK-TKIs, such as crizotinib [29]. Our subsequent results showed that when CQ was used in combination with lorlatinib, the phosphorylation level of Foxo3a was further reduced and the nuclear translocation was further enhanced. In vivo experiments also showed that when the phosphorylation of Foxo3a was strongly inhibited, the expression of Bim was significantly upregulated. From the above, lorlatinib induced autophagy through the PI3K/AKT/mTOR pathway. In contrast, the inhibition of autophagy would promote the dephosphorylation of Foxo3a and its nuclear translocation, thereby mediating the regulation of Bim transcription to promote apoptosis, which might be the way to exert an effect when autophagy inhibitors and lorlatinib work together. The regulatory effect of Foxo3a has been shown in the treatment of a variety of cancers. Possible mechanisms include the dephosphorylation of Foxo3a via the AKT-Foxo3a pathway that induces autophagy, through dephosphorylated Foxo3a, which increases the expression of pro-apoptotic proteins through nuclear translocation, or by the use of certain drugs that promote Foxo3a nuclear translocation through the AMPK/Foxo3a pathway, thereby promoting cell apoptosis. These results indicated that loarlatinib induced autophagy induced to protect cells from apoptosis, dephosphorylated Foxo3a, and promote Foxo3a nuclear translocation is the key link between inhibiting autophagy and promoting apoptosis. Our results suggested that enhanced autophagy was related to reducing the cytotoxicity of lorlatinib, and inhibition of autophagy may be an effective way to enhance the cytotoxicity of lorlatinib and delay the occurrence of drug resistance.

This study had several limitations. Currently, our group has reported that lorlatinib treatment induced autophagy in lung cancer cells in vitro and in xenograft models. At present, there are no effective therapies against lung cancer in the clinic other than treatment with CQ. It is urgent to find more and better clinically usable autophagy inhibitors in the future. Meanwhile, there have been related studies on the relationship between targeted therapy and autophagy, but the results are not exactly the same as those presented here. Crizotinib treatment of ALK+ anaplastic large cell lymphoma induced autophagy as a survival response, and excessive autophagy was related to cell death [30]. Therefore, more research is needed to clarify the effect of autophagy on the efficacy of ALK+ targeted therapy.

In summary, the acquired mechanisms of resistance to lorlatinib with ALK-rearranged lung cancers can be diverse and complex. We have demonstrated for the first time that a progressively higher level of protective autophagy caused by lorlatinib could decrease the cytotoxicity of lorlatinib. When CQ was used in combination with lorlatinib, the autophagy could be inhibited effectively by activating the Foxo3a/Bim axis and promoting Foxo3a nuclear translocation. Our study provides a new strategy through targeting autophagy to enhance lorlatinib cytotoxicity and further supports the exploration of combination strategies in clinical trials for patients with off-target resistant mechanisms.

## Materials and methods

### Cell lines and cell culture

H3122 and H2228 cells were purchased from the Shanghai Cell Bank (Shanghai, China). All cell lines were tested mycoplasma-negative. Both of these cell types were cultured in RPMI-1640 (Hyclone) with Earle’s salts, supplemented with 10% fetal bovine serum (FBS; Gibco), 2 mmol/L L-glutamine (Gibco), 100 U/mL penicillin (HyClone), and 100 µg/mL streptomycin (Hyclone) at 37 °C, with 5% CO_2_ and 90% humidity.

### Materials

Lorlatinib was purchased from Selleck Chemicals, and chloroquine (CQ) was purchased from Sigma-Aldrich. These were dissolved in dimethyl sulfoxide (DMSO; Sigma-Aldrich) at a final concentration of 1 mM and then stored at −80 °C. Hoechst 33342 was purchased from Sigma-Aldrich. Anti-LC3 (#12741S), SQSTM1/p62 (#8025S), Bim (#2933S), ALK (#3663), phosphorylated (Tyr1507) ALK (#14678), AKT (#9272S), phosphorylated (S473) AKT (#4060S), p-mTOR (#5536), mammalian target of rapamycin (mTOR; #2983), Foxo3a (#12829), P-Foxo3a (#9466), BCL-XL (#2764), P-MCL-1(#14765), MCL-1(#94296), and GAPDH (#2118S) antibodies were purchased from Cell Signaling Technology.

### Cell growth assays

Cell proliferation was assessed using a CCK-8 assay. Briefly, 2 × 10³ cells/well were seeded in 96-well plates and treated with lorlatinib or DMSO for 24 h. The treatment time was limited to <48 h. Then, 10 μL of CCK-8 in phosphate-buffered saline (PBS) was added to each well, and the plates were then incubated at 37 °C for 4 h. Next, the absorbance was measured at 450 nm using a plate reader (Bio-Rad Laboratories, Hercules, CA). All experiments were repeated at least three times.

### Colony-formation assays

Briefly, 1000 cells were resuspended in a culture medium and seeded in 6-well plates. After a series of concentrations of lorlatinib was added to cells for 14 days, the cells were fixed with 4% paraformaldehyde and stained with 0.1% crystal violet. After decolorization with 33% acetic acid, the sample’s absorbance was measured at 450 nm using a plate reader (Bio-Rad Laboratories, Hercules, CA). Triplicate samples were used in this experiment.

### Apoptosis assays

Cellular apoptosis was measured using flow cytometric analysis. The cells were collected by trypsinization after 48-h treatment with lorlatinib, washed three times with PBS, and then resuspended at a density of 1 × 10^7^ cells/mL. After double staining using the FITC Annexin V Apoptosis Detection Kit I and PI for 30 min at ambient temperature in the dark, the cells were analyzed using a flow cytometer (Beckman Coulter Novios, FL, USA). Acridine orange/ethidium bromide (AO/EB) fluorescence staining was also used to identify cell apoptosis as was previously described [31]. After 48-h treatment with lorlatinib and/or CQ, the cells were washed with PBS, stained with AO/EB solution (100 μg/mL, 1:1; Solarbio, Beijing, China), and then immediately used for fluorescence microscopy.

### Western blot analysis

Cells were collected by scraping, washed twice with PBS, and then lysed for 30 min at 4 °C in RIPA buffer (Sigma-Aldrich, France). After centrifugation at 12,000 × *g* for 20 min at 4 °C, the protein content was determined using a BCA assay. An equal amount of protein was loaded for gel electrophoresis for 2 h at 110 V, followed by transfer onto PVDF membranes (90 min, 200 mA) (Roche, Switzerland). These membranes were then blocked using 5% bovine serum albumin (BSA) for 1 h at room temperature and incubated overnight at 4 °C with primary antibodies. Subsequently, the membranes were washed and incubated with 0.02 µg/mL horseradish peroxidase-conjugated goat anti-rabbit (Sino Biological, China) for 1 h and visualized using the ChemiDoc Touch System (Bio-Rad, USA).

### Measurement of autophagic activity

The autophagic activity was monitored using the Cyto-ID Green Autophagy Detection Kit (Enzo Life Sciences, ENZ-51031-K200, ENZO, Farmingdale, NY, USA). The Cyto-ID Green autophagy dye served as a selective marker of autolysosomes and early autophagic compartments. Cells were washed with Assay Buffer, using 100 μL of Microscopy Dual Detection Reagent to cover each sample of monolayer of cells, and the cells were then protected from light and incubated for 20 min at 37 °C, after which they were then incubated with cyto-ID Green Detection Reagent for 30 min at room temperature. Afterward, 10,000 events/sample were analyzed by fluorescence microscopy. These six-well plates were imaged on an ImageXpress Micro (Molecular Devices) high-throughput imager. Image analysis was performed using the MetaXpress software.

### Transmission electron microscopy

Cells were seeded in 6-well plates and treated with lorlatinib for 2 days, and then these cells were fixed with 2.5% glutaraldehyde in 0.1 M PBS (pH 7.4) for 1 h at room temperature, followed by fixation with 1% osmium tetroxide for 1 h on ice. The samples were dehydrated step-wise into increasing concentrations of ethanol (50, 70, and 100%) and acetone and finally embedded in Araldite. Then, 60-nm sections were prepared using an LKB-I ultramicrotome, transferred to copper grids, and stained with uranyl acetate and lead citrate. Electron microscopy images were obtained using a Gatan JEM-1400 plus transmission electron microscope.

### In vivo PC xenograft tumor model

All of the experiments involving animals were approved by the Ethics Committee of the Army Medical University. The animals were maintained in individual ventilated cages in compliance with institutional guidelines. After subcutaneous injection of 1 × 10^6^ H3122 cells on the left side of 6-week-old female BALB/c A-nu mice (Laboratory Animal Center of Army Medical University, Chongqing, China), we randomly divided the mice into four groups (six mice/group). After the inoculation sites formed tumors about 50 mm^3^ in size within 5–7 days, the four groups were, respectively, established as the control, CQ treatment, lorlatinib treatment, and combined lorlatinib and CQ treatment groups. Based on other prior studies, the mice were given lorlatinib (1 mg/kg), CQ (2.5 mg/kg), or a combination of lorlatinib and CQ by means of intragastric administration per day [12, 31]. The size of the tumor was measured using a vernier caliper. The tumor sizes and body weights were recorded twice a week. The volume of tumors was calculated as follows: *V* = (length × width2)/2. Randomization and single blinding were used for measurement. The animals were monitored for 4 weeks until euthanasia, at which time the tumors were removed and weighed. The changes in the LC3 content were determined using immunohistochemistry.

### Statistical analysis

Statistical analysis was performed using GraphPad Prism 8.0, and the data are presented as the mean ± S.E.M. Two-tailed Student’s *t* test was performed to calculate significance in an interval of 95% confidence, and a value of *P* < 0.05 was considered to be statistically significant.

## Supplementary information

Supplemental Material

supplementary figures1,2,3

## Data Availability

All data generated or analyzed during this study are included in this article. The datasets used and/or analyzed during the current study are available from the corresponding author on reasonable request.
