# Supplementary material for: Protective autophagy decreases lorlatinib cytotoxicity through Foxo3a-dependent inhibition of apoptosis in NSCLC
Source: Cell Death Discov. 2022 Apr 22;8:221. doi: 10.1038/s41420-022-01027-z (PMC9033765; doi:10.1038/s41420-022-01027-z)
Supplement: Supplementary file 1 — Supplemental Material [file 41420_2022_1027_MOESM1_ESM.pdf]

Source data for Figure 1C

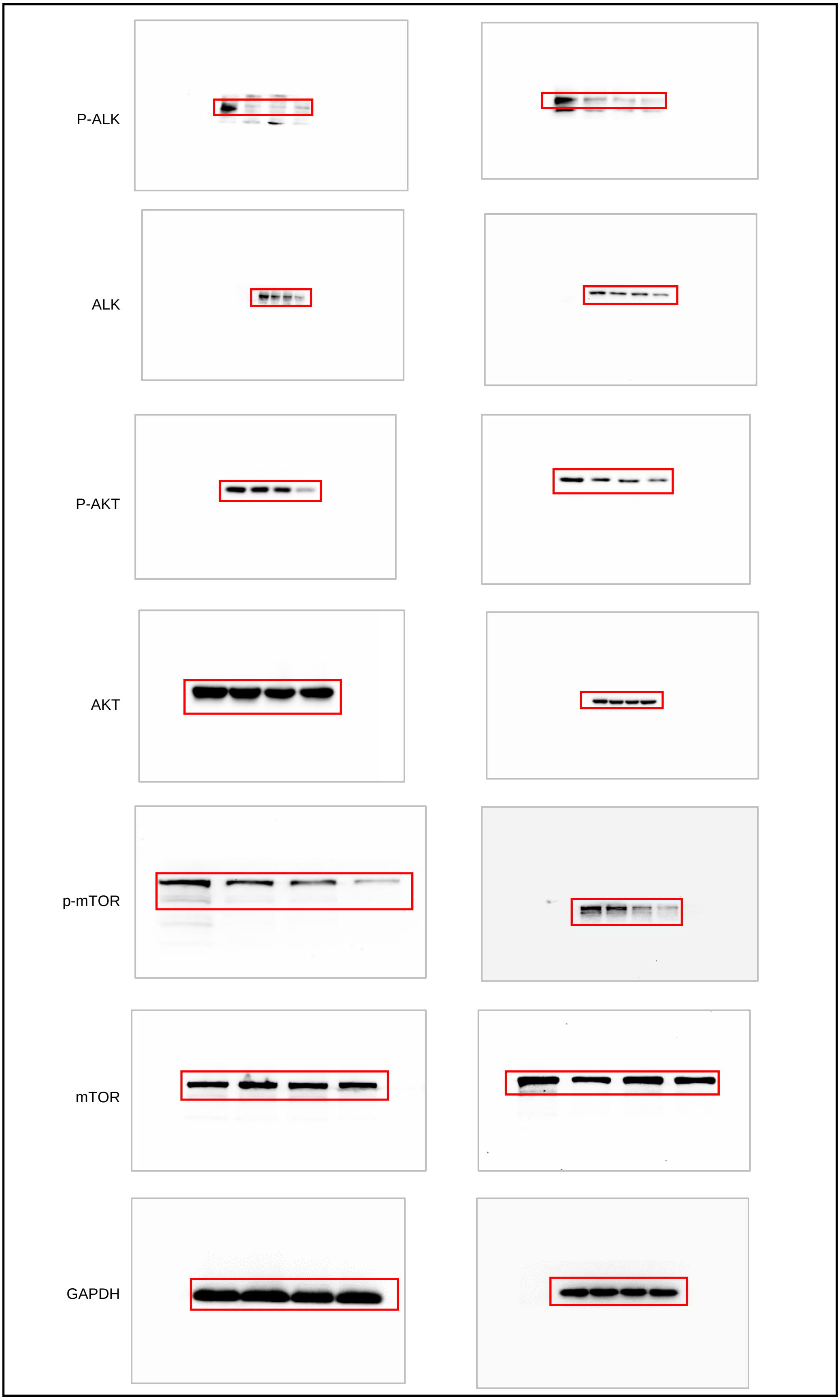

Full gel image related to the indicated figures.(continued on next page)

Source data for Figure 1F

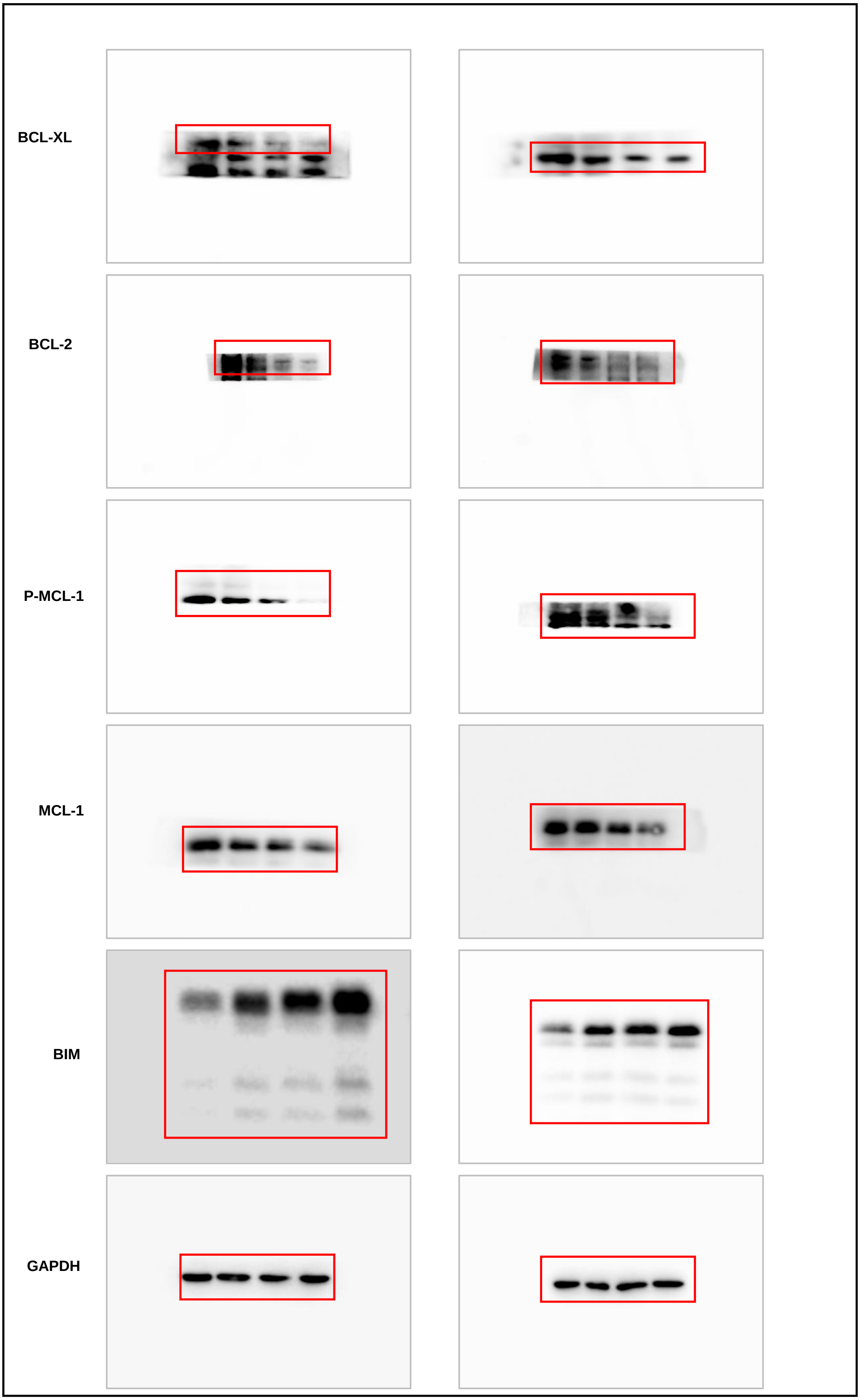

Full gel image related to the indicated figures. (continued on next page)

Source data for Figure 2C

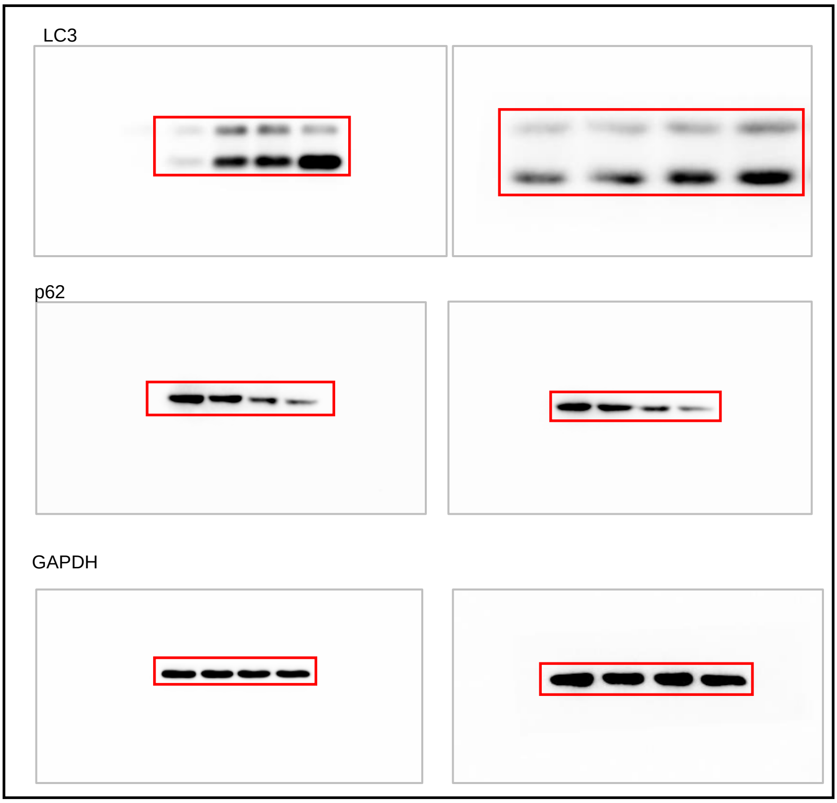

Source data for Figure 2D

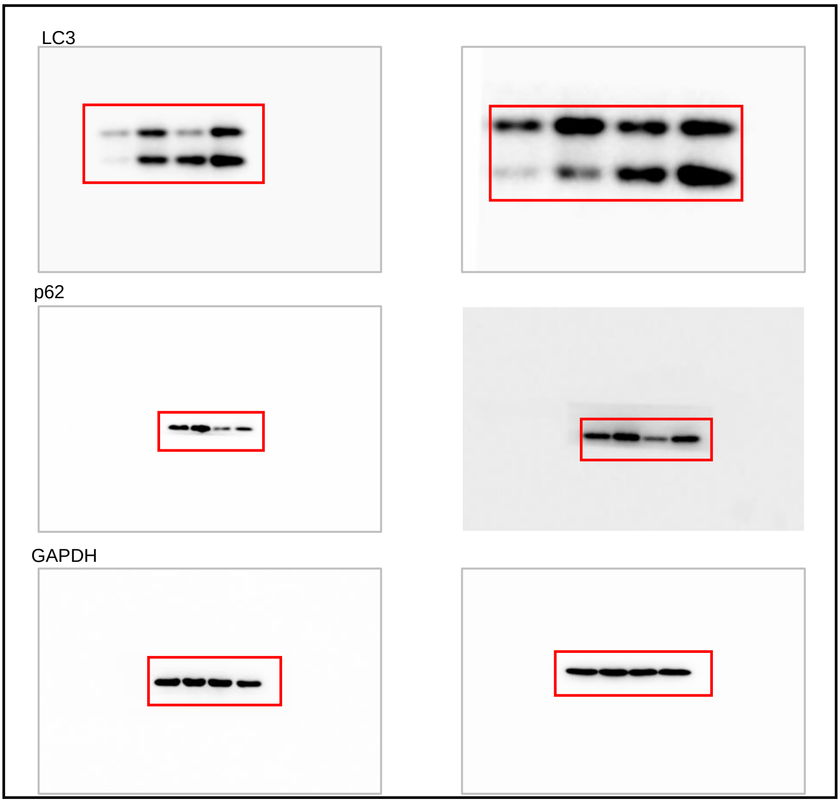

Full gel image related to the indicated figures.(continued on next page)

Source data for Figure 3A

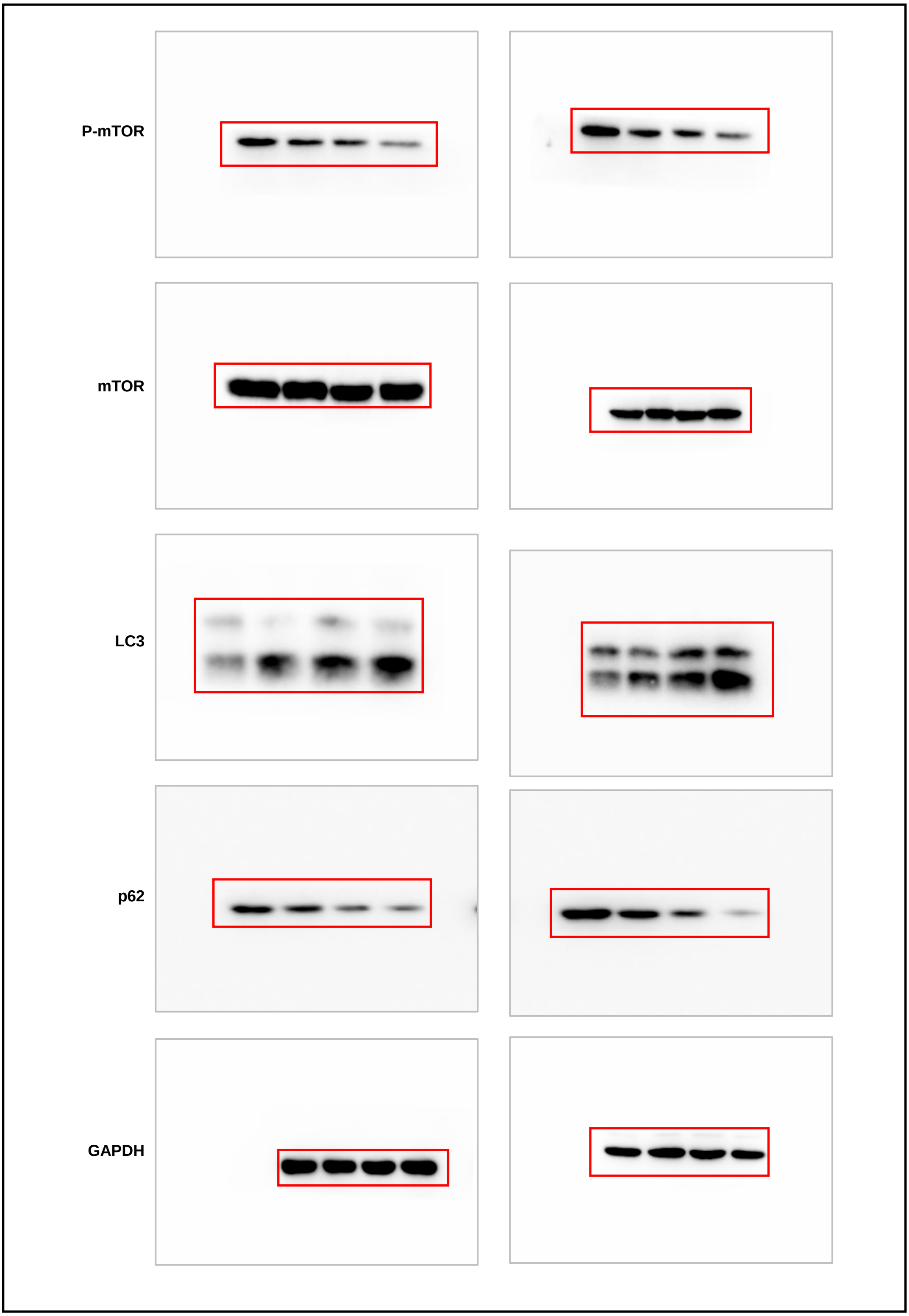

Full gel image related to the indicated figures. **(continued on next page)**

Source data for Figure 3B

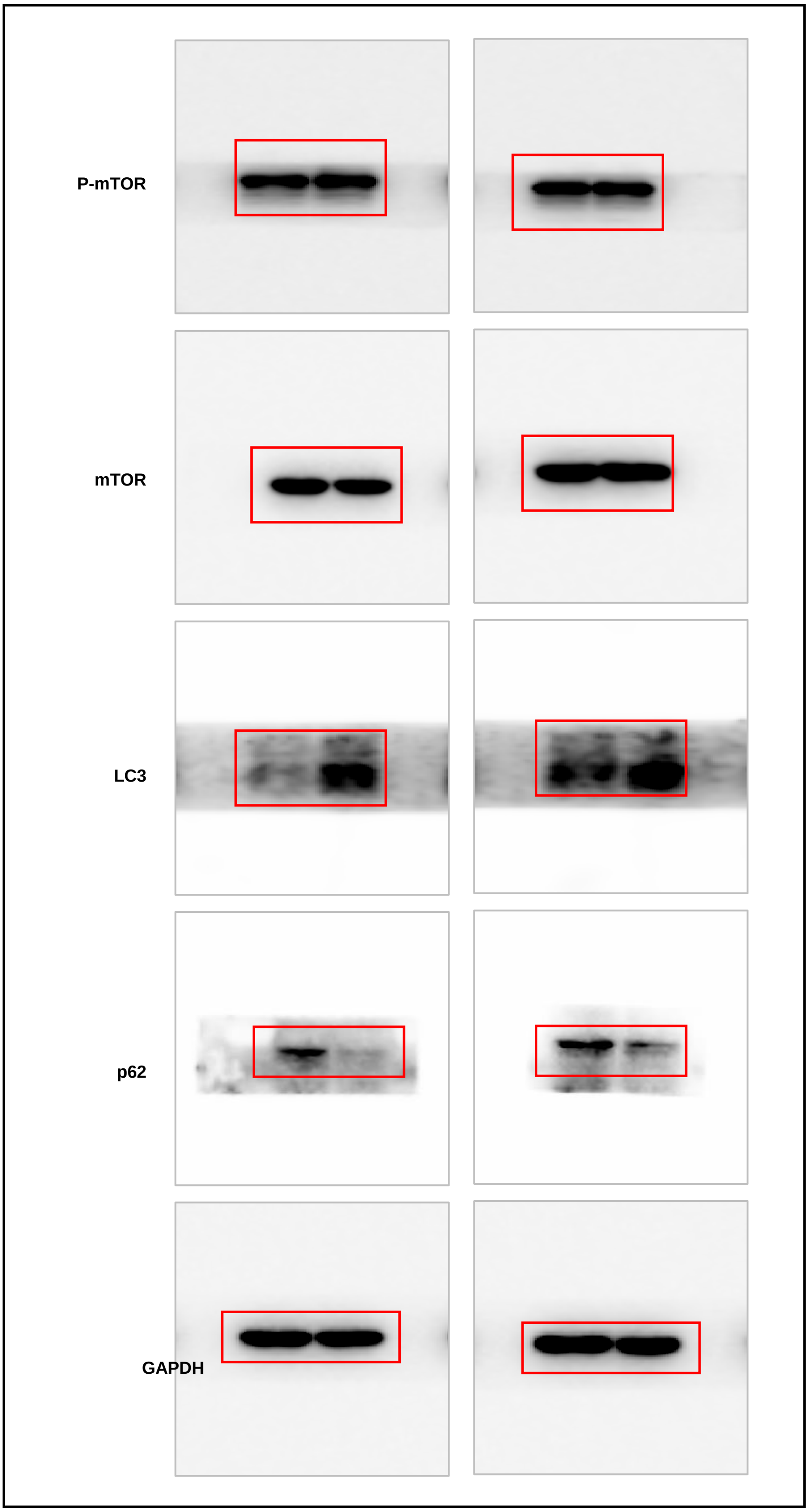

Full gel image related to the indicated figures. **(continued on next page)**

Source data for Figure 3F

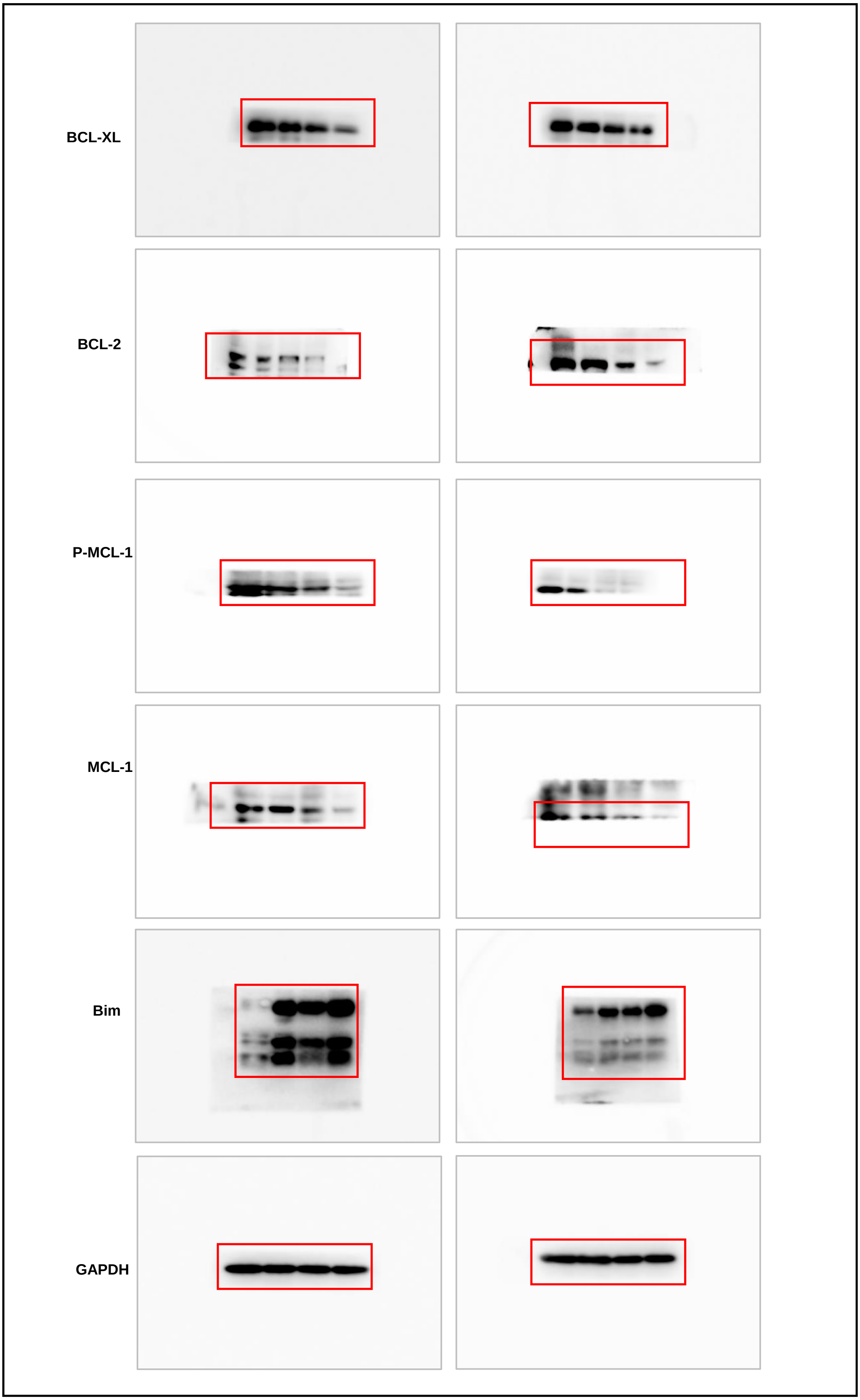

Full gel image related to the indicated figures. **(continued on next page)**

Source data for Figure 4A

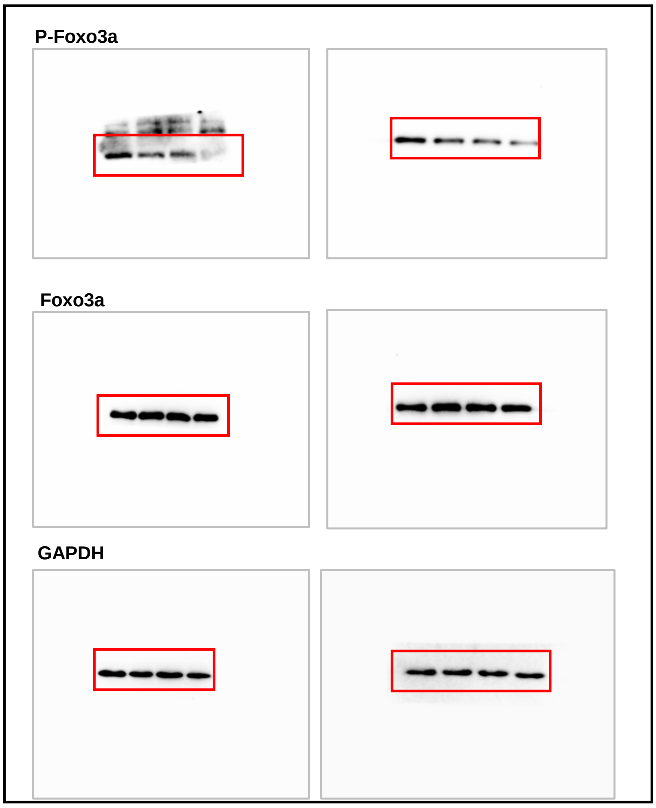

Source data for Figure 4B

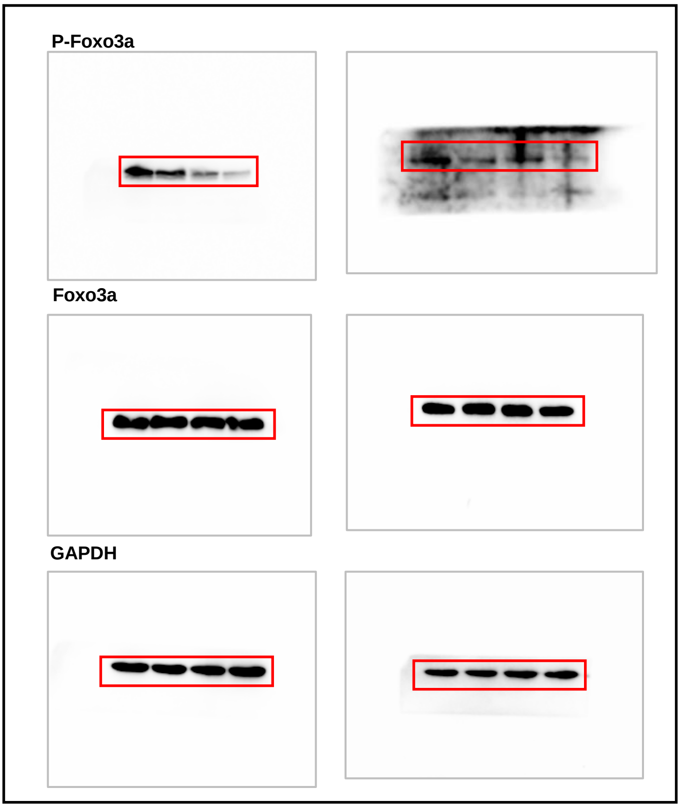

Full gel image related to the indicated figures. (continued on next page)

Source data for Figure 4D

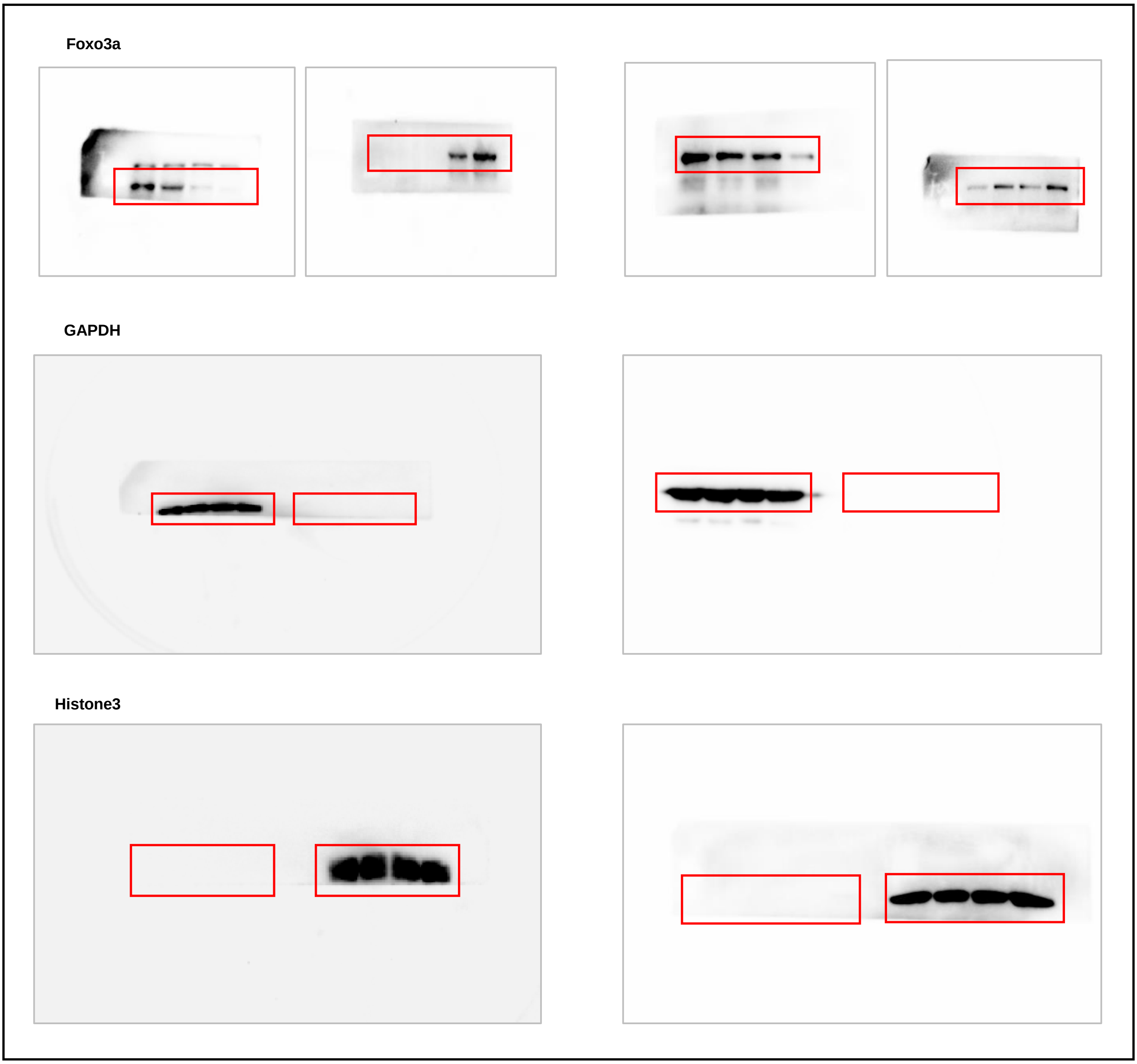

Full gel image related to the indicated figures. (continued on next page)

Source data for Figure 5G

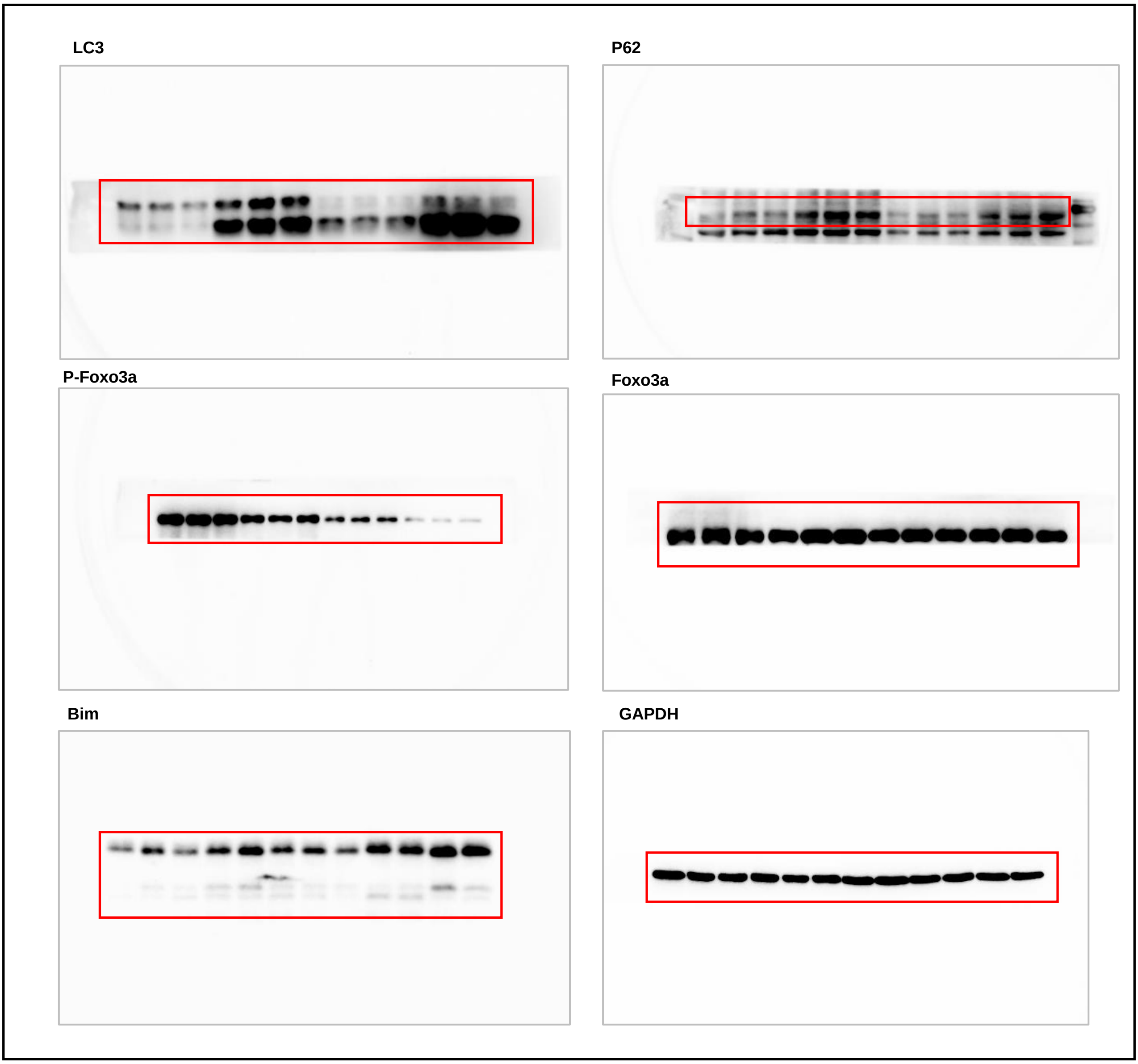

Full gel image related to the indicated figures. **(continued on next page)**

Source data for Figure S3B

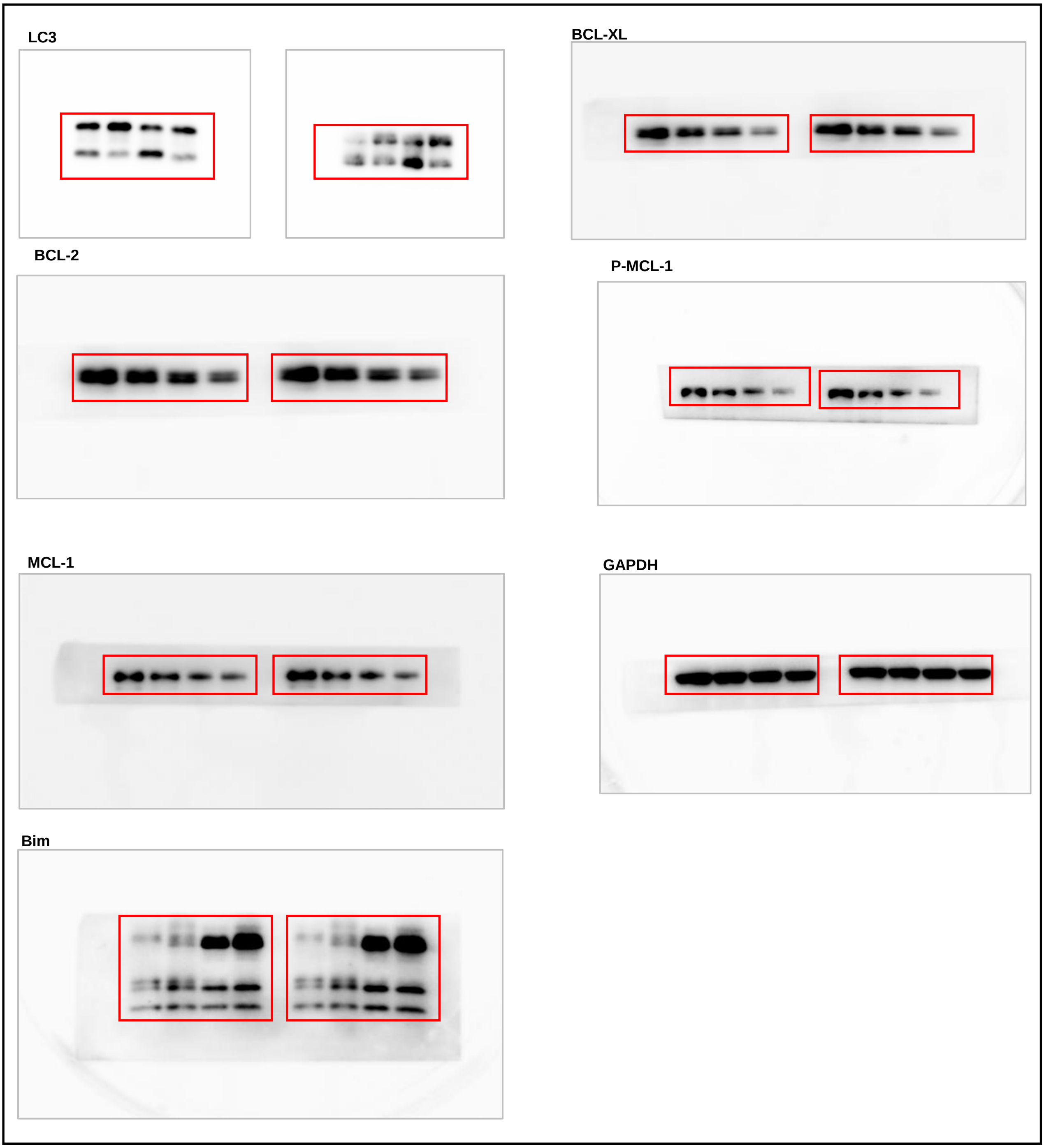

Full gel image related to the indicated figures
