# Supplementary material for: Protective autophagy decreases lorlatinib cytotoxicity through Foxo3a-dependent inhibition of apoptosis in NSCLC
Source: Cell Death Discov. 2022 Apr 22;8:221. doi: 10.1038/s41420-022-01027-z (PMC9033765; doi:10.1038/s41420-022-01027-z)
Supplement: Supplementary file 2 — supplementary figures1,2,3 [file 41420_2022_1027_MOESM2_ESM.pdf]

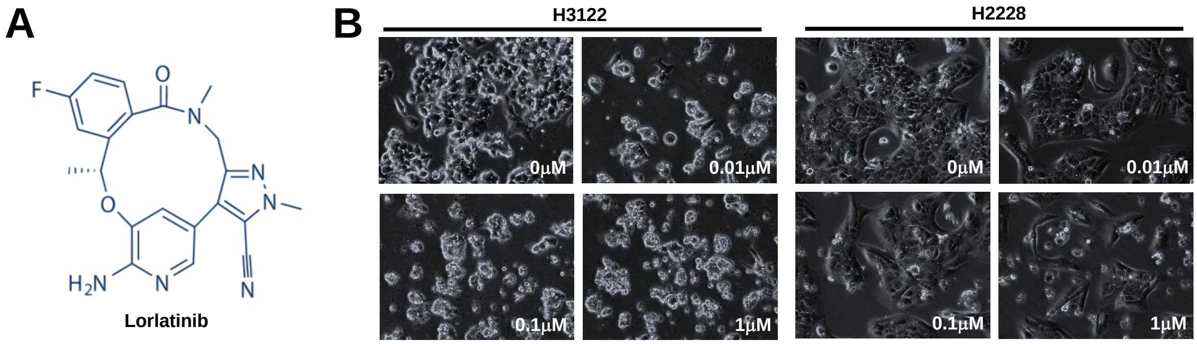

Supplemental Figure 1. (A) Chemical structure of lorlatinib. (B) Micrographs of cells treated with lorlatinib as indicated dose for 48 h.

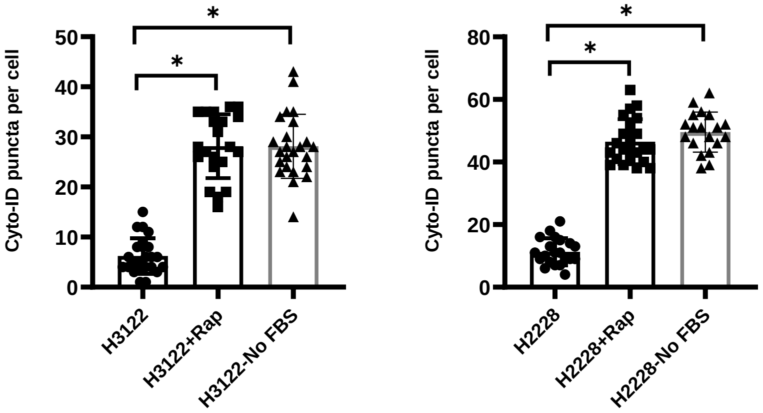

Supplemental Figure 2. The quantification and statistical analysiss were shown the autophagosomes of H3122 and H2228 cells which were treated with Rap (500nM) or serum deprivation. Rap:rapamycin. *,p<0.01

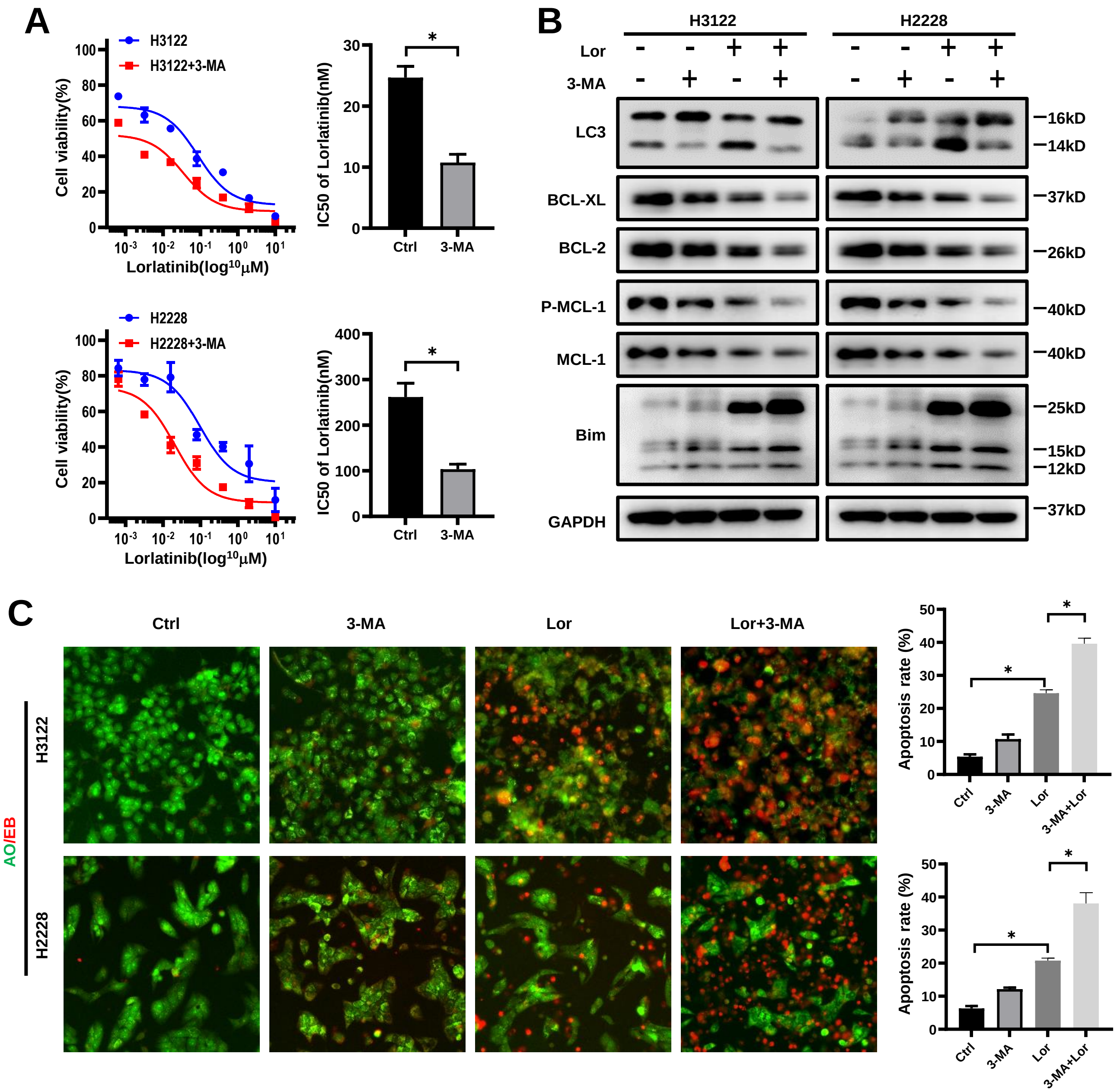

Supplemental Figure 3. Lorlatinib-induced autophagy protects cells from apoptosis. (A) Cell viability cck-8 assay for cells treated with the indicated concentrations of lorlatinib with or without 3-MA(100µM) for 48 h. (B) Cells were treated by Lorlatinib(10nM) with or without 3-MA (100µM) for 48 h. Western blot analysis was performed to the indicated antibody. GAPDH was included as a loading control. (C) Cells were treated by Lorlatinib(10nM) with or without 3-MA (100µM) for 48 h, then stained with AO/EB. Lor, lorlatinib. *,$p<0.01$
